# Self-Guided Digital Intervention for Depression in Adolescents: Feasibility and Preliminary Efficacy Study

**DOI:** 10.2196/43260

**Published:** 2023-11-22

**Authors:** Ian Miller, Emily Peake, Gabriel Strauss, Elise Vierra, Xin Koepsell, Brandon Shalchi, Aarthi Padmanabhan, Jessica Lake

**Affiliations:** 1 Limbix Health, Inc San Francisco, CA United States; 2 Big Health Inc San Francisco, CA United States

**Keywords:** depression, adolescents, young adults, cognitive behavioral therapy, behavioral activation, digital health, mobile interventions, mobile phone

## Abstract

**Background:**

Depression in adolescents is a large and growing problem; however, access to effective mental health care continues to be a challenge. Digitally based interventions may serve to bridge this access gap for adolescents in need of care. Digital interventions that deliver components of cognitive behavioral therapy (CBT) have been shown to reduce symptoms of depression, and virtual reality (VR) may be a promising adjunctive component. However, research on these types of treatments in adolescents and young adults is limited.

**Objective:**

This study aims to evaluate the feasibility, acceptability, and preliminary efficacy of Spark (v1.0), a 5-week, self-guided, CBT-based digital program using a mobile app and VR experiences to target symptoms of depression in adolescents.

**Methods:**

A single-arm, open-label study of the Spark program was conducted with a community sample of 30 adolescents and young adults aged 12 to 21 years with self-reported moderate to severe depression symptoms. Participants completed a weekly depression assessment (Patient Health Questionnaire-8) in the app during the 5-week intervention period as well as web-based baseline, postintervention, and 1-month follow-up self-report assessments. The participants also completed a qualitative postintervention interview. For participants aged <18 years, caregivers completed assessments at baseline and postintervention time points. Feasibility outcomes included recruitment rate (the proportion of participants who enrolled in the study divided by the total number of participants screened for eligibility) and retention rate (the proportion of participants who completed postintervention assessments divided by the total number of participants who received the intervention). Acceptability outcomes included engagement with the program and quantitative and qualitative feedback about the program. Preliminary efficacy was evaluated based on the Patient Health Questionnaire-8.

**Results:**

The study recruitment (31/66, 47%) and retention (29/30, 97%) rates were high. Participants provided higher ratings for the ease of use of the Spark program (8.76 out of 10) and their enjoyment of both the mobile app (7.00 out of 10) and VR components (7.48 out of 10) of the program, whereas they provided lower ratings for the program’s ability to improve mood (4.38 out of 10) or fit into their daily routines (5.69 out of 10). We observed a clinically and statistically significant reduction in depression scores at postintervention (mean difference 5.36; *P*<.001) and 1-month follow-up (mean difference 6.44; *P*<.001) time points.

**Conclusions:**

The Spark program was found to be a feasible and acceptable way to deliver a self-guided CBT-focused intervention to adolescents and young adults with symptoms of depression. Preliminary data also indicated that the Spark program reduced the symptoms of depression in adolescents and young adults. Future studies should evaluate the efficacy of this intervention in an adequately powered randomized controlled trial.

**Trial Registration:**

ClinicalTrials.gov NCT04165681; https://classic.clinicaltrials.gov/ct2/show/NCT04165681

## Introduction

### Background

Depression is the second most common mental health disorder among adolescents and young adults and is a critical health problem in the United States [1,2]. It is also the leading cause of mental health–related disease burden globally, with 34% of adolescents and young adults reporting depressive symptoms [3,4]. The rate of depression has been rising sharply, with a recent 5-year increase of 63% [5], and suicide is the second-leading cause of death among adolescents and young adults [2,5,6]. These alarming trends highlight an urgency to develop effective and accessible treatments [1].

Despite the high prevalence rates of depression among adolescents and young adults, access to effective mental health care is often limited by a number of factors. First, concerns over privacy or stigma result in limited treatment seeking and discontinuation of psychotherapy [7-10]. Second, the demand for mental health professionals has outstripped supply, particularly in rural areas, which is a problem that is predicted to intensify over the next 10 years [11,12]. At the same time, waitlists for mental health providers are often at least 2 to 6 months long and represent one of the most substantial barriers to receiving care [11-14]. Finally, parents of children with mental health concerns also perceive financial burden as a significant barrier to accessing care [15]. Consequently, up to 60% to 80% of adolescents and young adults with depression do not receive appropriate or timely treatment [16].

Digital interventions may serve as an effective, accessible, and acceptable solution to these problems [17]. Accumulating evidence highlights the efficacy of digital health interventions for mental illness [18-21]. As digital health interventions can be completed at home, they may reduce stigma-related barriers to treatment, leading to higher rates of engagement and self-disclosure [22,23], and increase treatment accessibility if access to mental health providers or resources is limited [21]. Given the scalability of digitally delivered interventions, these programs offer a more affordable treatment option than traditional therapies. Digital treatments also hold particular promise for adolescents and young adults as youth report high levels of smartphone use [24] and enjoy using novel technologies [17]. Finally, patients report digital health interventions to be acceptable and empowering tools that can facilitate self-understanding and personal change [23-25].

Cognitive behavioral therapy (CBT) is well established as effective in the prevention and treatment of depression in children and adolescents [26]. CBT is a psychosocial intervention focused on the relationship between thoughts, feelings, and behaviors and on teaching coping skills to overcome symptoms [27,28]. Preliminary data suggest that digital forms of CBT can be effective in the treatment of anxiety and depression in youth [20,21,26,29], although the available evidence in adolescents and young adults is limited. Behavioral activation (BA) is a component of CBT as well as a stand-alone treatment that specifically encourages participation in personally meaningful activities that are rewarding or bring about a sense of mastery [30-36]. It includes assessment and progress toward personal goals through motivational strategies, reward seeking, natural reinforcers, self-monitoring, skills training, and reduction of maladaptive coping behaviors that may occur during depressive episodes [30]. Adolescents and young adults may be especially susceptible to reward-related deficits because of immaturities in reward-processing neural systems, impacting their ability to both experience reward and overcome avoidance [37,38]. In fact, the behavioral aspects of CBT may be the mechanism of action through which CBT reduces symptoms of depression and have been shown to be as effective as cognitive approaches in reducing depressive symptoms in youth [30,34,36,39]. Therefore, a self-guided mobile BA program could be a promising opportunity for the treatment of depression in adolescents and young adults [29,40,41].

Virtual reality (VR) holds potential as a modality for delivering behavioral interventions because it is designed to simulate naturalistic environments and present controlled and ecologically valid situations and contexts. VR exposure therapy, wherein patients are virtually exposed to the things they fear, equivalently reduces psychological symptoms compared with in vivo exposure in patients with anxiety, phobias, and posttraumatic stress disorder [42-44].

VR treatments have also been effectively delivered at home and may reduce the burden on clinician time, particularly in areas where mental health resources are limited or in high demand [45,46]. Nevertheless, to date, there have been few investigations of VR treatments for depression, and the feasibility of VR as a modality for depression treatment has not been established.

Most digital interventions for depression in adolescents have been computerized in nature, but other technologies such as mobile and VR may be well suited for delivering self-guided digital health interventions [40,41]. Given that lower-income families are more likely to have a mobile device than a computer at home [47], a mobile-based program may be more accessible to a wider group of adolescents and young adults. The portability of a mobile device may also allow for more flexibility in completing program activities, integrating more easily into an individual’s daily routine, and potentially increasing treatment adherence rates, which have typically been low in self-guided interventions [48]. At the same time, although accessibility may be a limitation of VR, the immersive nature of this technology has the potential to increase engagement with a fully self-guided program [49]. VR has also been shown to effectively change attitudes and behaviors that generalize to the real world [50-53], likely because of the ecological validity and immersiveness of VR experiences, which could enhance the impact of a digital health intervention.

Spark v1.0 is a novel, 5-week, self-guided BA-based program for depression in adolescents delivered through mobile and VR technology. The content of the Spark program was developed in consultation with clinical subject matter experts and is based on established BA protocols [31,33].

### Objectives

This study aimed to assess the feasibility, acceptability, and preliminary evidence of the efficacy of the Spark program. Given the familiarity with and frequent use of technology within the target population, it was anticipated that this program would be feasible and acceptable for adolescents and young adults with depressive symptoms.

## Methods

### Overview

A single-arm, open-label clinical trial design was implemented, with all participants receiving the 5-week Spark program (ClinicalTrials.gov; NCT04165681). The study was conducted between October 2019 and April 2020. Participants completed baseline and postintervention visits in person and a 1-month follow-up questionnaire via email. Given the onset of the COVID-19 pandemic, 3 participants completed the postintervention visit via videoconference.

### Recruitment

Participants were recruited from the greater Los Angeles area, California, via paid internet advertisements, community flyers, and local media. A total of 31 adolescents and young adults aged 12 to 21 years with self-reported depression symptoms were enrolled in this study. Initial eligibility was determined using a prescreen. Participants or their caregivers (if participants were aged <18 y) expressed interest by providing their contact information and then completing a brief phone-based prescreening with study staff to assess initial eligibility. If participants were found to meet initial eligibility based on the phone screen, they were emailed a screening questionnaire consisting of the Patient Health Questionnaire-8 (PHQ-8) [54] and a question to confirm that they were not pregnant to complete the eligibility screening process.

### Eligibility Criteria

Inclusion criteria were a score of ≥10 on the PHQ-8, English fluency and literacy, access to a mobile device, participant willingness to provide informed consent or assent, and having a caregiver willing to provide informed consent if the participant was aged <18 years.

Exclusion criteria included seizure disorder or other neurological disorders; prior diagnosis of or currently receiving treatment for a substance use disorder, bipolar disorder, or psychosis; hospitalization or residential or inpatient treatment for a suicide attempt or self-harming behaviors within the past 3 months; current diagnosis of a reading or learning disability or intellectual disability; currently receiving treatment (including but not limited to medication or psychotherapy) for a cognitive disorder, including attention deficit/hyperactivity disorder; significant vision or hearing impairment; participation in a concurrent treatment study; self-reported pregnancy; and history of significant motion sickness.

If the participants were ineligible, they or their caregivers were notified of their ineligibility and provided with a list of national and local mental health resources.

### Procedure

Participants aged ≥18 years provided written consent to participate. Participants aged <18 years provided written assent to participate, and their caregivers provided written consent. After obtaining consent, all participants were assigned to complete the 5-week Spark program. Participants completed baseline assessments and received VR equipment and an initial orientation to the Spark program, which included training on how to set up and use the VR headset and how to download and use the mobile app. Caregivers of participants aged <18 years also completed baseline assessments. Participants were shown how to enable the use of Wi-Fi on the device and were asked to connect the device to their home Wi-Fi to remotely track the VR headset activity in the event that a headset was not returned at the end of study participation. However, access to a Wi-Fi connection for remote tracking was not required for participation, as the VR activity was also logged locally on a secure digital card within the VR headset. Weekly PHQ-8 assessments were completed in the mobile app. During the baseline visit, postintervention site visits were scheduled for between 5 and 7 weeks after the baseline site visit. At the postintervention site visit, participants and caregivers completed the postintervention assessments. Participants also completed an audio-recorded qualitative interview with a research team member about their experience of using the program. The study team (JL, XK, and AP) conducted postintervention interviews with participants to collect qualitative feedback on their experience with the Spark program.

If scheduling a postintervention site visit for between 5 and 7 weeks after baseline was not possible, participants were sent postintervention assessments via email to complete at home so that only the postintervention interview was completed on site outside of the time window. The participants had access to the mobile app and VR experiences until they returned for their postintervention study visit. To assess willingness to continue using the program, participants were asked at the postintervention site visit whether they would like to continue using the VR headset or mobile app until the 1-month follow-up time point. They could choose to continue using either the VR headset or mobile app or to continue using both. If they did not indicate that they wanted to continue using the mobile app or VR headset at this time, their access to the mobile app was restricted, and they were asked to return the VR headset.

One month after the postintervention site visit, participants who had elected to continue using the VR headset or the mobile app were asked to return the VR headset and had their access to the mobile app restricted.

Participants were compensated in the form of a gift card worth US $25 per hour for each site visit and US $25 for completing the 1-month follow-up survey. They also received US $15 bonus if they completed all 5 weekly in-app PHQ-8 assessments on time and US $50 for returning the VR headset at the end of the study. Participants were compensated US $12.50 for the completion of the postintervention assessments if they were completed at home; otherwise, completion was compensated as part of the US $25 per hour for the site visit. Participants could earn up to US $130 for meeting all these milestones. Compensation was not contingent on the completion of the mobile program or VR experience. The caregivers were not compensated for their participation.

### Safety Monitoring

During the course of data collection and monitoring, the research staff consulted with study clinicians if (1) patient symptoms declined to a clinically significant degree (an increase in PHQ-8 score ≥5 points from week to week) or (2) if a participant reported anything concerning in a free response in the app, in an assessment, or while directly communicating with the study staff. The principal investigator and study clinicians reviewed any clinical concerns in relevant assessments, free responses, and spontaneous reports of participants to the study staff to assess whether it was appropriate for the participant to continue study participation and whether further action to ensure the safety and well-being of the participant was warranted.

### Ethical Considerations

This study was reviewed and approved by the Western Copernicus Group Institutional Review Board (approval ID: WIRB protocol #20201686) and was registered on ClinicalTrials.gov (NCT04165681). Potential participants were told that their participation was voluntary, that they were free to discontinue participation at any time, and that all information would be kept confidential, with the exception of (1) mandatory limits to confidentiality and (2) safety concerns for them or others. Study data were deidentified and stored on Health Insurance Portability and Accountability Act–compliant cloud-based servers.

### Program Description

The Spark v1.0 program was developed in close collaboration with clinical psychologists, who guided decisions based on clinical protocols [31,33] and clinical experience, and with adolescents and young adults in co-design sessions to ensure that the program met user needs and resonated with the target users. The app consisted of 5 modules designed to be completed 1 module per week for 5 weeks, although modules could be completed at the user’s own pace. Each module contained mobile app components and 1 companion VR experience. Both the mobile app and VR experiences could be accessed without an internet connection after the initial download. Within the mobile app, participants read text on the screen, answered multiple-choice style questions, input free-form text responses, swiped or clicked buttons to progress through screens, dragged and dropped elements on the screen, indicated responses with a slider bar, and listened to audio clips. The mobile app had 2 tabs: modules and tools. The modules tab contained the content of the 5 program modules that progressed in a linear fashion, that is, each step in a module had to be completed to progress to the next step, and the user had to complete an entire module before moving onto the next module. The tools tab contained on-demand resources including mood tracking, guided meditation audio, a list of the VR experiences accessible in the VR headset, and emergency resources for patients experiencing thoughts of suicide or self-harm. The structure of the program is outlined in Table 1. Detailed information about the Spark program is provided in Multimedia Appendix 1 [31,55-58].

**Table 1:** The structure of the Spark program.

App module and VR^a^ experience number	Mobile app modules	Corresponding VR experience
1	Introduction to the program, depression, and behavioral activation	Immersive video about depression and behavioral activation (eg, the connection between thoughts, feelings, and actions)
2	Mood tracking, identifying the relationship between mood and behavior, and growth mindset	Educational immersive video clip about the relationship between thoughts, feelings, actions, and neural plasticity
3	Introduction to mindfulness and activity scheduling	Mindful breathing exercise
4	Introduction to problem-solving and continued activity scheduling	Guided meditation about problem-solving
5	Mindfulness, relapse prevention, and continued activity scheduling	Guided meditation about transformation and relapse prevention

^a^VR: virtual reality.

A Pico Interactive Goblin VR headset, leveraging a proprietary VR software platform, was used to deliver the VR content. Users could explore their virtual environment by moving their head. In some VR experiences, elements of the environment could be modified or selected using gaze-controlled selection, meaning an individual could look at a particular part of the environment for a certain duration to initiate selection. The VR headsets were set up to log when a participant entered and exited a virtual environment. The durations of some VR experiences varied, as there were elements of user interaction and exploration that impacted the time spent within the experience.

### Measures

#### Feasibility

Assessment of the feasibility of the study included evaluating a CONSORT (Consolidated Standards of Reporting Trials) diagram and calculating the recruitment rate (the proportion of those who enrolled in the study divided by the total number of individuals screened for study eligibility) and retention rate (the proportion of those who completed the postintervention assessments divided by the total number of participants who received the intervention). The percentage of VR headsets returned and the percentage of participant data lost because of not enabling remote tracking of VR headset activity were also evaluated as part of the study feasibility.

#### Adolescent Self-Report Measures

##### Overview

The schedule of assessments is presented in Table 2.

**Table 2:** Participants’ and caregivers’ schedule of assessments.

		Baseline	Weekly during intervention	Postintervention follow-up	1-mo follow-up
**Adolescent-reported measures**					
	Baseline questionnaire	✓			
	PHQ-8^a^	✓	✓	✓	✓
	CES-D^b^	✓		✓	✓
	GAD-7^c^	✓		✓	✓
	PANAS-C^d^	✓		✓	✓
	BADS-SF^e^	✓		✓	✓
	SF-20^f^	✓		✓	✓
	Postprogram questionnaire			✓	
**Caregiver-reported measures**					
	MFQ-PS^g^	✓		✓	
	Postprogram questionnaire for caregivers			✓	

^a^PHQ-8: Patient Health Questionnaire-8.

^b^CES-D: Center for Epidemiological Studies Depression Scale.

^c^GAD-7: 7-item Generalized Anxiety Disorder Scale.

^d^PANAS-C: Positive and Negative Affect Schedule for Children.

^e^BADS-SF: Behavioral Activation for Depression Scale–Short Form.

^f^SF-20: 20-item Short Form Survey.

^g^MFQ-PS: Moods and Feelings Questionnaire–Short Parent version.

##### Baseline Questionnaire

A 12-item self-report questionnaire administered at baseline included questions about demographics (eg, age, gender, ethnicity, and race) and prior and current treatment for depression and other mental health disorders, and it asked participants to rate their expectations of treatment benefit from the Spark program on a 0 (completely unhelpful) to 10 (completely helpful) Likert scale. This questionnaire was developed internally to characterize the participant sample.

##### PHQ-8 Instrument

The PHQ-8 [54] is an 8-item self-report measure for screening for depression and for establishing depression severity. The PHQ-8 has been demonstrated to have high sensitivity and specificity in adolescents and young adults. The total score ranges from 0 to 24, with a higher score indicating greater depression symptom severity. The PHQ-8 was administered at prescreening, baseline, postintervention, and 1-month follow-up time points as well as weekly during the intervention period.

##### Center for Epidemiological Studies Depression Scale

The Center for Epidemiological Studies Depression Scale (CES-D) [59] is a 20-item screening tool for depression and other depressive disorders. It has good sensitivity, good specificity, and high internal consistency. Scores range from 0 to 60, with a higher score indicating greater depressive symptoms. The CES-D was administered at baseline, postintervention, and 1-month follow-up time points.

##### 7-Item Generalized Anxiety Disorder Scale

Given the high comorbidity between anxiety and depression, we assessed anxiety symptoms using the 7-item Generalized Anxiety Disorder Scale (GAD-7) [60]. The GAD-7 is a brief 7-item assessment for generalized anxiety disorder with satisfactory sensitivity and specificity for the most common anxiety disorders encountered in primary care settings [61]. The total score ranges from 0 to 21, with a higher score indicating greater anxiety symptom severity. The GAD-7 was administered at baseline, postintervention, and 1-month follow-up time points.

##### Positive and Negative Affect Schedule for Children

The Positive and Negative Affect Schedule for Children (PANAS-C) [62] is a widely implemented measure consisting of 30 items assessing respondents’ emotions for the past few weeks. The items are grouped into 2 subscales: positive affect and negative affect. Scores on the positive affect subscale range from 12 to 60. Scores on the negative affect scale range from 15 to 75. Higher scores on the positive affect subscale indicate greater endorsement of positive emotions, and higher scores on the negative affect subscale indicate greater endorsement of negative emotions. The PANAS-C was administered at baseline, postintervention, and 1-month follow-up time points.

##### Behavioral Activation for Depression Scale–Short Form

The Behavioral Activation for Depression Scale–Short Form (BADS-SF) [63] is a 9-item scale designed to measure changes in avoidance and activation along the course of BA therapy for depression. The total scores range from 0 to 54, with higher scores indicating higher activation. The BADS-SF was administered at baseline, postintervention, and 1-month follow-up time points.

##### 20-Item Short Form Survey

The 20-item Short Form Survey (SF-20) [64] is designed to measure overall physical and mental health. Scores are transformed to a scale from 0 to 100, with higher scores indicating better health status. The SF-20 was administered at baseline, postintervention, and 1-month follow-up time points.

##### Postintervention Questionnaire

An internally developed 23-item self-report questionnaire administered at the postintervention time point included questions about current treatment for depression and other mental health disorders, changes in treatment for a mental health condition after baseline assessment, and undesirable side effects of the program. The questionnaire asked participants to rate how much they thought the program improved their mood or symptoms of depression, quality of life, and coping skills on a 10-point Likert scale (0=not at all and 10=completely). It also included questions evaluating participant experience with the program as a whole (eg, enjoyment, ease of use, willingness to continue using, willingness to recommend to a friend, and helpfulness of mobile and VR experiences) and questions seeking feedback on each module separately, including whether participants would be disappointed if VR was removed from each module. These items were also rated on a 10-point Likert scale. Other survey questions captured product development–related information that are beyond the scope of this study and therefore not reported here.

#### Caregiver-Reported Measures

These measures were administered to caregivers of participants aged <18 years.

##### Moods and Feelings Questionnaire–Short Parent Version

The Moods and Feelings Questionnaire–Short Parent version (MFQ-PS) [65] consists of a series of 13 descriptive phrases regarding how the participant has been feeling or acting recently and is a screening tool for depression in children and young people. The total score ranges from 0 to 26, with higher scores indicating greater symptom severity. Caregivers completed the MFQ-PS at baseline and postintervention time points.

##### Postintervention Questionnaire for Caregivers

An internally developed 21-item questionnaire was administered at the postintervention time point with free-response, multiple-choice, and Likert-scale questions about demographics, impressions of the impact of the Spark program on their child, willingness to have their child continue using the Spark program, and willingness to refer the Spark program to a friend whose child was experiencing depression symptoms.

##### Quantitative Engagement Metrics

Mobile app engagement metrics evaluated at the end of the intervention period included the number of participants completing all app modules, the number of participants completing each individual app module, the mean and range of mood tracking entries entered, and the mean and range of BAs scheduled and completed within the 5-week intervention period. Module completion was defined as completing all required tasks in a module, as captured by mobile app analytics. BA completion was defined as participants marking whether they had completed a scheduled activity, as described in the Multimedia Appendix 1.

A priori VR engagement metrics included the number of participants completing all VR experiences and the number of participants completing each individual VR experience. Owing to limitations in the programming of the VR analytics, it was not possible to determine whether VR experiences were completed, in part because the duration of the VR experiences varied with user interactivity. Therefore, the number of participants who entered a VR experience and the average time spent on each VR experience were reported instead.

The number of participants who chose to continue using the mobile app or VR headset until the 1-month follow-up appointment and the number of participants who actually continued to use the mobile app or VR headset during this 1-month period were evaluated.

##### Qualitative Feedback

The study staff asked the same questions addressing major objectives, with the potential for individualized follow-up questions. Topics included how the Spark program fit into participants’ daily schedule and affected their symptoms, its ease of use, feedback on the VR and mobile app components of the program, whether they liked using the product and would recommend it to a friend, and whether they felt the content was relevant to them. Feedback on what aspects of the program they thought could be improved was also collected.

### Data Analysis

#### Feasibility and Acceptability

A CONSORT diagram was created, and descriptive statistics for recruitment and retention rates were calculated as part of establishing feasibility. Acceptability of the intervention included descriptive statistics of engagement metrics from the app and VR, average ratings from postintervention surveys, and qualitative evaluation of postintervention interviews. Interviews were conducted and recorded, and detailed interview notes were taken by the study staff (JL, XK, and AP). A grounded theory approach was used to analyze the data from the postintervention interviews to derive emergent themes related to the acceptability of and engagement with the Spark program [66]. Themes were generated through an inductive process of coding involving iteratively categorizing and sorting data through pattern recognition [67,68]. Interview notes were reviewed, iteratively coded, and grouped into themes until consensus on key themes was reached (IM, EP, and BS).

#### Preliminary Efficacy

Preliminary efficacy was evaluated based on PHQ-8 scores and secondary outcomes. Using multiple imputation in an intention-to-treat sample, missing PHQ-8 data at the item and time point levels were imputed with 100 data sets. A CI approach using multiple levels of confidence to evaluate the strength of preliminary evidence was used to evaluate whether the mean treatment difference (baseline to postintervention and baseline to 1-mo follow-up) was >0, and whether CIs included a clinically meaningful difference [69,70]. Clinically meaningful difference was defined as an average reduction in PHQ-8 scores of ≥5 points between baseline and postintervention time points [69,71]. Treatment response and remission rates were calculated at the postintervention and 1-month follow-up time points. Treatment response was defined as a postintervention PHQ-8 score of <10 and 50% lower than the baseline PHQ-8 score [72]. Remission was defined as a postintervention score of <5 [72,73].

An exploratory general linear mixed model was conducted with random effects (intercepts only) for both participants and items and a fixed effect of time (weeks 0-5 and week 9) to evaluate the statistical significance of the change in PHQ-8 scores with time. Within-subjects 2-tailed *t* tests were conducted to evaluate the statistical significance of the change in PHQ-8 scores between (1) baseline and postintervention time points, (2) baseline and 1-month follow-up time points, and (3) postintervention and 1-month follow-up time points.

Means and SDs for secondary outcomes (CES-D, GAD-7, PANAS-C, BADS-SF, SF-20, and MFQ-PS) at baseline, postintervention, and 1-month follow-up time points as well as mean differences and 95% CIs for baseline versus postintervention time points and baseline versus 1-month follow-up time points are presented in Multimedia Appendix 2.

## Results

### Demographics and Participant Characteristics

A total of 30 participants received the study intervention. Refer to Table 3 for participant characteristics. The sample recruited was racially diverse, and 70% (21/30) of the participants were female, consistent with higher rates of depression and treatment seeking in adolescent girls [74,75]. The average PHQ-8 score at baseline was 14.93, which is considered moderate severity. Most participants (19/30, 63%) were receiving concurrent treatment (medication, psychotherapy, or both) for symptoms of depression. The expected treatment benefit of the Spark program at baseline, as assessed via the baseline questionnaire, was rated as moderate (mean 6.47, SD 2.03) on a scale of 0 to 10, with 0 being completely unhelpful and 10 being completely helpful.

**Table 3:** Baseline characteristics (N=30).

Characteristic		Value
Age (y), mean (SD)		17.03 (2.97)
**Gender, n (%)**		
	Male	8 (27)
	Female	21 (70)
	Nonbinary	1 (3)
**Ethnicity, n (%)**		
	Hispanic or Latino	6 (20)
**Race, n (%)**		
	Asian	6 (20)
	Black	3 (10)
	White	10 (33)
	Mixed race	7 (23)
	Unknown	4 (13)
Baseline PHQ-8^a^ score, mean (SD)		14.93 (3.92)
**Concurrent treatment for depression, n (%)**		
	Medication only	2 (7)
	Therapy only	10 (33)
	Medication and therapy	7 (23)
	None	11 (37)
Expectation of treatment benefit, mean (SD)		6.47 (2.03)

^a^PHQ-8: Patient Health Questionnaire-8.

### Feasibility and Acceptability

**Figure 1:**
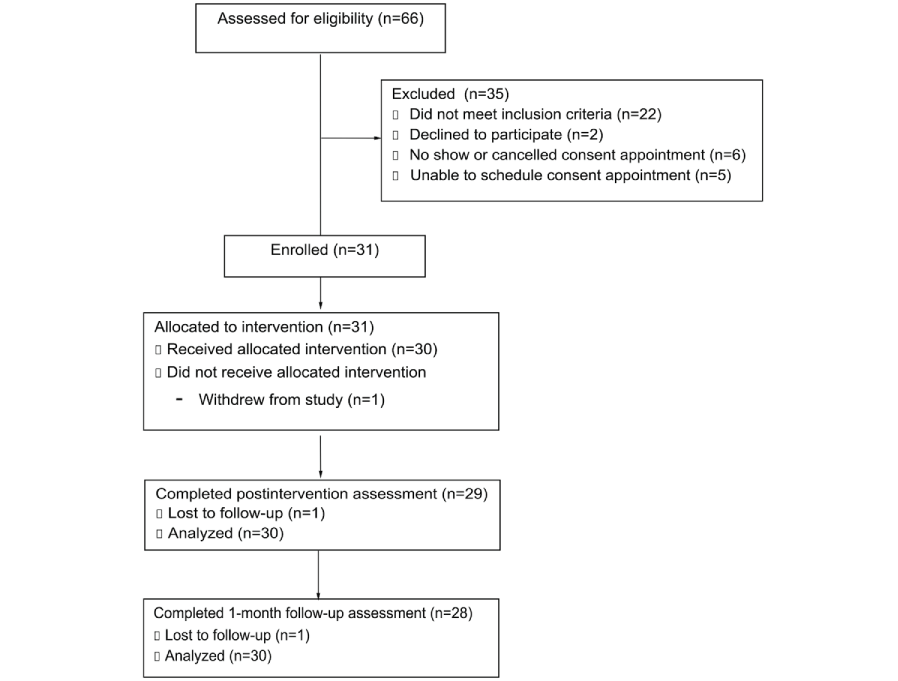
CONSORT (Consolidated Standards of Reporting Trials) diagram.

A total of 66 participants were screened for eligibility. Of these 66 participants, 22 (33%) did not meet the eligibility criteria; 2 (3%) declined to participate; and 11 (17%) either did not show, canceled their appointment, or were unable to schedule a consent appointment. A total of 31 adolescents (and their caregivers when required) were enrolled in the study, representing a recruitment rate of 47%. One participant withdrew from the study before receiving the program, resulting in a final sample of 30 participants who received the program. A high retention rate of 97% (29/30) was observed at the postintervention time point, with only 1 participant lost to follow-up before the postintervention assessment. Another participant was lost to follow-up before the 1-month follow-up time point. Refer to Figure 1 for the CONSORT diagram. A total of 8 participants were unable to return for their postintervention site visit within 7 weeks after the baseline site visit and were therefore sent and completed postintervention assessments at home and completed the postintervention interview on site when available to do so. All participants except 1 (29/30, 97%) returned the VR headsets, but logged VR data were available for all participants because of enabled remote logging of activity over Wi-Fi.

### Mobile App and VR Engagement

#### Mobile App

All mobile app data were retrievable at study conclusion. The module completion rates are presented in Table 4. In total, 50% (15/30) of the participants completed all 5 mobile app modules within the 5-week intervention period. During the 5-week intervention period, participants completed an average of 6.7 (SD 4.48; range 0-14) mood logs. Participants scheduled an average of 3.17 (SD 2.31; observed range 0-8) BAs and completed an average of 2.37 (SD 2.06; observed range 0-7) BAs. The average BA completion rate was 71% (23/30) for participants who scheduled at least 1 BA.

**Table 4:** Mobile app and virtual reality (VR) experience completion rates by the end of 5 weeks (N=30).

Module number	Estimated time to complete VR experience (min)	Mobile app module completion, n (%)	Entered VR experience, n (%)	Time spent in VR exercise, mean (SD)
1	25	27 (90)	27 (90)	15 min 36 s (10 min 43 s)
2	2	27 (90)	26 (87)	1 min 55 s (47 s)
3	10	23 (77)	26 (86.7)	9 min 40 s (9 min 22 s)
4	7	19 (63)	25 (83.3)	4 min 22 s (2 min 45 s)
5	12	15 (50)	22 (73.3)	6 min 39 s (4 min 46 s)

Notably, relative to the 50% of participants who completed the intervention by the end of the 5-week intervention period, an additional 10 participants (25/30, 83%) completed all the mobile app modules before their postintervention study visit date, despite there being no requirement to complete the program before the study visit or compensation tied to program completion.

Of the 29 participants who returned for a postintervention visit, 18 (62%) endorsed that they would like continued access to the mobile app until the 1-month follow-up time point. However, of these 18 participants, 4 (22%) progressed in the app (eg, completed additional mood logs, psychoeducational tasks, or scheduled or completed BAs) during that period.

#### VR App

Table 4 reports the number of participants who entered each VR experience and the average duration of time they spent in each experience.

At their postintervention visit, of the 30 participants, 5 (17%) expressed interest in continued access to the VR headset until the 1-month follow-up time point. Of these 5 participants, 2 (40%) continued to use the device during that period.

### Postintervention Questionnaire Feedback

Table 5 summarizes the participant and caregiver feedback on the mobile app modules and VR experiences. Overall, participants reported a high ease of use of the Spark program and highly rated their enjoyment of both the mobile app and VR components. Participants gave moderate ratings for program helpfulness, how the program improved their coping skills and fit into their routine, and their willingness to recommend the program to a friend. Caregivers of adolescents aged <18 years rated their children’s coping skills as moderately improved, consistent with adolescent ratings. Alternatively, caregivers rated the program more highly than adolescents in terms of ease of fitting into a routine, their willingness for their child to continue using the app, and their willingness to recommend the program to a friend. Both caregivers and adolescents rated the program’s impact on improving mood and quality of life within the mild to moderate range.

**Table 5:** Postintervention questionnaire feedback.

Question (all questions on a scale of 0-10, where 0=not at all and 10=completely)	Adolescents (n=29), mean (SD)	Caregivers (n=18), mean (SD)
How much do you feel like Spark improved your or your child’s mood or symptoms of depression?	4.38 (2.14)	5.22 (2.32)
How much do you feel like Spark improved your or your child’s quality of life?	4.38 (2.44)	4.83 (2.57)
How much do you feel like Spark improved your or your child’s coping skills?	5.31 (2.83)	5.18 (2.70)
How easy was it to include Spark in your or your child’s daily routine?	5.69 (2.79)	7.11 (2.61)
How willing would you be to continue or have your child continue using Spark to manage your depression?	6.00 (2.95)	8.39 (1.94)
How willing would you be to recommend Spark to a friend who or whose child is feeling down or who you think may be depressed?	6.10 (2.51)	7.94 (1.92)
How easy was it to use Spark?	8.76 (1.68)	N/A^a^
Overall, how enjoyable was Spark?	6.90 (2.32)	N/A
How enjoyable did you find the virtual reality experiences during Spark?	7.48 (1.86)	N/A
How enjoyable did you find the mobile app experience during Spark?	7.00 (2.27)	N/A
How helpful did you find the virtual reality experiences during Spark?	6.21 (2.53)	N/A
How helpful did you find the mobile app experiences during Spark?	6.41 (2.77)	N/A

^a^N/A: not applicable.

In total, 17% (5/30) of the participants reported experiencing undesirable side effects from using the Spark program in the postintervention questionnaire, all of which were concerning the VR experiences (ie, 3 reported headaches, 1 reported feelings of dissociation or unreality, and 1 reported dizziness and eye pain).

### Postintervention Interview: Emergent Themes

#### Overview

A total of 27 participants participated in postintervention interviews. Qualitative analysis of the postintervention interviews revealed key themes of gamification, digital therapeutic alliance, personalization, deriving insights, and proactive versus reactive use related to the acceptability of and engagement with the Spark program.

#### Gamification and Interactivity

Gamification is the process of adding game-like qualities and interactivity to nongame systems, with the goal of increasing user engagement. Overall, participants reported desiring more gamification and interactivity in both the mobile app and VR experiences of the program. For example, some participants suggested including smaller games within the mobile app or making the material more interactive. Some participants indicated that the program contained too much passive reading of or listening to content.

#### Digital Therapeutic Alliance

The theme of digital therapeutic alliance was identified, which was defined during data analysis as the bond between a digital guide and a user composed of shared bonds, goals, and tasks. A digital therapeutic alliance is considered an important component of mobile-based interventions [76,77]. Overall, participants reported feeling a therapeutic bond with the digital character guiding them through the mobile app, which allowed them to feel a sense of ownership of their own progress. For example, participants reported feeling attended to by the program and like someone was watching out for them, despite the program being self-guided. Others reported being reminded of a role model (eg, a therapist or teacher) because the program guide helped them review the activities to ensure understanding. Alternatively, some participants suggested that the program could have provided more personalized and relatable support. Others felt that the program ended abruptly, which led them to feel left on their own.

#### Personalization

The theme of personalization was identified, which was defined during data analysis as the degree of user control in the activities of the program and the flexibility to move through the program. This theme was specific to the mobile app portion of the program. Generally, participants reported enjoying the personalization and flexibility incorporated into the mobile app. For example, some participants reported enjoying the values, customization of BAs, and free-response portions of the mobile app because it gave them a feeling of autonomy. Participants also indicated a desire for more personalization. For example, participants requested the opportunity to personalize the mobile app home page and the program guide and for changes to be made to the app based on their behavior.

#### Deriving Insights

The theme of deriving insights was identified, which was defined during data analysis as the identification of common patterns and mastering skills to apply to one’s personal situation. This theme was specific to the mobile app portion of the program. Participants reported that the active therapeutic components of the program (eg, mood tracking and BAs) made them feel like they had more control of their mood, helped them structure their time, and provided concrete choices. Features such as mood tracking and activity scheduling also helped the participants make sense of their emotional progress. For example, participants felt that mood tracking provided insights into their behaviors and understanding of the types of situations or activities that made them feel better or worse. Participants similarly favored activity scheduling because it helped them set a plan, and participants felt that they could cope with new challenges.

Some participants noted that adding additional tools and skills to the app could lead to confusion about what and how to implement these skills in the real world. One participant mentioned that during a depressive episode, it could be difficult to think clearly and identify an appropriate coping strategy. However, overall, deriving insights helped participants feel like they had more control of their experience with the app and increased their sense of self-efficacy.

#### Proactive Versus Reactive Use

The final theme identified was proactive versus reactive use. Proactive use was defined as learning and practicing skills in preparation for when challenges arise. Reactive use was defined as turning to the program as a coping response to a stressor, for example, to calm down after a recent event. Analysis showed that participants differentially used the mobile app versus VR experiences. Specifically, participants often turned to the mobile app in a proactive way, for example, to learn new coping skills that they could implement in the real world to cope with their depression. Alternatively, participants described turning to VR more often in a reactive way to improve their negative mood in the moment. For example, using it after school to “destress,” to “calm down” during stressful episodes, or in the moment for “quick relief.”

### Preliminary Efficacy

**Figure 2:**
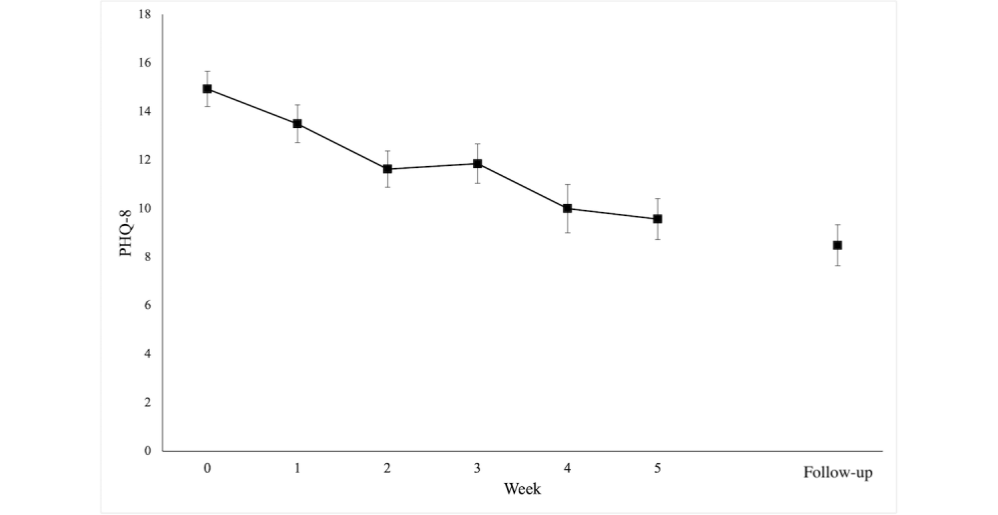
Patient Health Questionnaire-8 (PHQ-8) scores by week with standard error bars.

**Figure 3:**
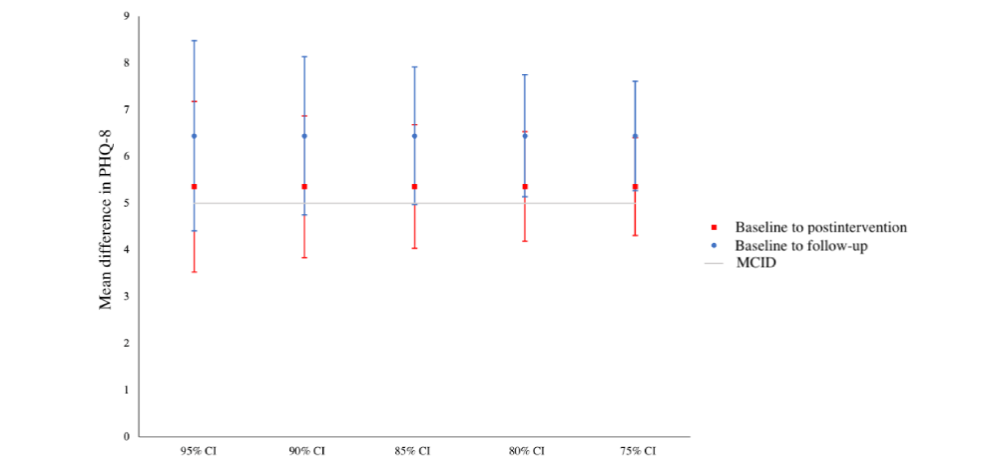
Multiple CIs for baseline to postintervention time point and baseline to 1-month follow-up time point differences in Patient Health Questionnaire-8 (PHQ-8) scores. MCID: minimal clinically important differences.

Weekly reported depressive symptoms (Figure 2) declined along the course of the 5-week intervention (Δbaseline – postintervention = 5.36) and continued through the 1-month follow-up (Δbaseline – 1-mo follow-up = 6.44). A CI approach demonstrated that all levels of confidence included a clinically meaningful difference in PHQ-8 scores at the postintervention timepoint, and all levels of confidence either included or exceeded a clinically meaningful difference in PHQ-8 scores at 1-month follow-up (Figure 3). The general linear mixed model showed a significant main effect of time (*F*_1, 34775,435_=145.795; *P*<.001). Paired 2-tailed *t* tests showed a significant reduction in depressive symptoms from baseline to postintervention time point (t_29_=5.995; *P*<.001) and from baseline to 1-month follow-up (t_29_=6.46; *P*<.001) time point. The difference between the values at postintervention and 1-month follow-up time points was not significant (*P*=.24). At postintervention time point, the rate of treatment response was 36.67% and the rate of remission was 6.67%. At 1-month follow-up, the rate of treatment response was 43.33% and the rate of remission was 23.33%.

As presented in Multimedia Appendix 2, mean differences in secondary outcome measures between baseline and postintervention time point and between baseline and 1-month follow-up time point demonstrated improvements in depression and anxiety symptoms, positive and negative affect, daily activity and health perceptions, and caregiver-reported depression symptoms at 95% CIs.

## Discussion

### Overview

This study aimed to investigate the study feasibility, intervention acceptability, and preliminary evidence of the efficacy of Spark, a mobile app and a VR-based program for adolescents with depression. The results supported study feasibility, as demonstrated by the high recruitment, retention, and VR equipment return rates. Overall, both quantitative and qualitative data supported the acceptability of the treatment modalities to deliver self-guided therapeutic content for depression in adolescents. Improvements in depressive symptoms and secondary measures also provide preliminary evidence of efficacy of the program.

### Principal Findings

#### Feasibility and Acceptability

Study feasibility, as indicated by recruitment and retention rates, was notably high [78-80]. This may be, in part, because of flexible study procedures, which included allowing participants to complete certain aspects of the program remotely if they were not able to return in person within a specified time frame. In addition, at the start of the COVID-19 pandemic, all remaining study visits were completed through videoconferencing, without requiring an in-person postintervention visit, which also likely contributed to the high retention and points to the benefits of virtual trials [80,81]. Finally, although this information was not tracked formally, it was noted that recruitment was driven largely through advertising to caregivers rather than directly to adolescents, which may have led to a sample of participants with particularly engaged caregivers who contributed to high retention rates, for example, by facilitating transportation to study visits as needed.

The delivery of the intervention via the mobile app was feasible and acceptable. Participants reported high ease of use ratings, which is critical for a self-guided program. Having access to a mobile phone was a requirement for study enrollment, and no potential participants were excluded for this reason, suggesting that this was not a significant limitation in recruitment. This finding is consistent with data showing that 95% of adolescents nationally own or have access to a smartphone [82]. Nevertheless, although requiring access to a mobile phone was not a limitation in study recruitment because eligibility criteria were presented to potential participants before prescreening procedures, it is possible that some individuals were prevented from participating for this reason. Given that many schools now provide tablets to students [83], allowing the use of a tablet may facilitate study participation in the future, although privacy and security considerations would be necessary [84,85]. A strength of the mobile app was that it could be accessed offline, limiting any potential impact of regional differences in access to high-speed internet, while not resulting in any data loss because data analytics could be delivered once a user’s phone had reconnected to the internet. Future studies will need to evaluate the impact of using the Spark program offline on study data integrity in rural areas, as this study was conducted exclusively in an urban area.

Qualitative and quantitative data supported that, overall, participants found the mobile app enjoyable and helpful. Participants noted that they enjoyed certain aspects of the app such as gamification, interactivity, and personalization, which could serve to enhance learning transfer [84]. Half (15/30, 50%) of the study participants had completed all mobile app modules by the end of the intervention period, which is comparable with other digital health intervention studies [86,87]. Although engagement was promising for an early version of the product, greater program adherence should theoretically help drive better outcomes, as participants engage with more therapeutic content. Therefore, it is important to better understand the drivers of app engagement and disengagement. Understanding such underlying factors and developing features to support app engagement will be prioritized for further app development. Notably, the percentage of participants who had completed all the app modules increased to 80% (24/30) by the time participants returned for their postintervention site visit. This increase in program completion may simply have been because of the additional time available to complete the program between the end of the intervention period and the postintervention site visits. Alternatively, this increase in app completion may suggest feelings of accountability or obligation to complete the modules before participants returned for their postintervention site visit with study staff. This explanation is consistent with evidence that human support is an important factor in driving engagement in digital health interventions [88,89]. To prevent high dropout rates in real-world use, methods for incorporating human support into self-guided programs will be considered, for example, involving health care providers in monitoring app use and allowing for interaction with patients to encourage use and reward progress [88].

Overall, the use of the VR headset was feasible and acceptable, although limitations were observed. No participants reported difficulty using the VR headset or following instructions for use. The VR experiences were rated as enjoyable by the participants, and caregivers strongly endorsed that the VR components contributed to the success of the program. All VR headsets except for 1 were returned at the end of the intervention, validating the feasibility of providing equipment loans for the intervention and likely supporting the effectiveness of incentivizing the equipment’s return.

However, VR was associated with limitations in study feasibility and intervention tolerability. For example, not all participants enabled Wi-Fi on the headsets at home to allow remote tracking of headset use, limiting the researchers’ ability to track activity and encourage use, as appropriate, during the intervention period. In addition, although no participants reported difficulty using the VR headset, several participants reported adverse events pertaining to the use of the headset (eg, dizziness, headache, and dissociation from reality) at rates consistent with prior literature [53]. Coupled with cost and logistical barriers to treatment using VR technology, these limitations may temper enthusiasm for a VR-based intervention, especially if the intervention can be successfully administered through more accessible technology.

Although not the main objective of this study, some notable comparisons between the mobile app and the VR experiences were possible. First, qualitative interviews suggested that participants used the mobile app and VR components of the intervention differentially. VR was used more reactively when experiencing symptoms and allowed for greater immersiveness, whereas the mobile app was used more proactively to support skills learning and practice. Although the program structure across the 2 modalities likely contributed to this finding, the nature of VR versus mobile modalities may have predisposed them to be used in different ways. An intended advantage of VR was to help immerse users, which is thought to be an effective method to enhance therapy [49,53]. However, the immersiveness of VR limits the time and environment in which it can be practiced and may have impacted the ease of incorporating the program into daily routine. Alternatively, previous research suggests that mobile devices allow for greater flexibility in completing program activities because they can be more seamlessly integrated into people’s lives [48]. Second, more participants requested to continue using the mobile app than the VR headset between postintervention and 1-month follow-up time points. This observation is consistent with the reports of differential use of mobile and VR components and the fact that there are limits to the situations in which VR can be practiced, which may have made the VR experiences more difficult to incorporate into daily routines than the mobile app modules. Future studies should evaluate how to maximally take advantage of these different technologies to support symptom reduction.

#### Preliminary Efficacy

Weekly PHQ-8 scores demonstrated both statistically and clinically significant reductions in depressive symptoms, providing preliminary evidence for the efficacy of the Spark program. These data were supported by secondary measures of depression symptom reduction, including caregiver reports, as well as reductions in anxiety and negative affect. The secondary outcomes also showed improvements in positive affect and functional outcomes. Consistent with the PHQ-8 scores, caregivers and adolescents rated the program as having a mild to moderate impact on symptoms and quality of life. Findings also suggested that improvements in depressive symptoms were maintained at 1-month follow-up. Future studies are needed to replicate these findings in a larger sample and further evaluate how long improvements in symptoms are sustained after intervention.

### Limitations

This study has a few notable limitations. First, the conclusions regarding program efficacy are limited by a number of important factors, including the small sample size and lack of a control arm. Given that placebo effects are strong in this patient population [90], it is particularly important to conduct sham-controlled studies to provide convincing evidence of treatment benefits. In addition, confounding factors, such as concurrent treatment, were not accounted for in the analyses and may have contributed to the observed symptom reductions. As indicated previously, recruitment was largely driven by advertising to caregivers. This may also have biased the study sample toward a group of adolescents with more engaged caregivers and affected the results. Additionally, it is important to note that participants were compensated for attending the consent session, completing weekly questionnaires, and returning the VR headset, thus potentially affecting the recruitment and retention rates..

A couple of factors also limited our ability to make direct comparisons between the mobile app and VR experiences. First, because of the small sample size, it was not possible to determine the relative contributions of the mobile app or VR experiences to symptom reduction. Furthermore, limitations in the programming of VR analytics affected our ability to determine VR experience completion rates and to compare those rates with mobile app completion rates. Modifications would need to be made in the future to more accurately determine VR headset engagement and to directly compare the relative utility of mobile device versus VR as a delivery mechanism.

### Conclusions and Future Directions

This study of a self-guided, CBT-based mobile app and VR program targeting depression symptoms in adolescents provides early evidence of feasibility, acceptability, and efficacy. Future studies should evaluate the efficacy of this intervention in an adequately powered randomized controlled trial. It will also be worth further investigating whether the benefits of VR, including increased immersiveness and caregivers’ sense that VR contributed to program success, outweigh the limitations observed in VR feasibility and acceptability. The study results more clearly support that a mobile intervention is an acceptable modality for integrating a self-guided intervention for reducing symptoms of depression into the daily lives of adolescents, a population with high technological familiarity. Given the ubiquity of mobile app access and use among adolescents, a digital intervention may reduce logistic, economic, and stigma-related barriers associated with standard psychotherapy. An efficacious self-guided intervention to treat the symptoms of depression in adolescents could have an important impact on this growing mental health concern and provide an accessible solution that could mitigate the lack of available resources to address it.

## Appendix

Multimedia Appendix 1 Spark program description.

Multimedia Appendix 2 Secondary outcomes.

## Abbreviations

BA: behavioral activation
BADS-SF: Behavioral Activation for Depression Scale–Short Form
CBT: cognitive behavioral therapy
CES-D: Center for Epidemiological Studies Depression Scale
CONSORT: Consolidated Standards of Reporting Trials
GAD-7: 7-item Generalized Anxiety Disorder Scale
MFQ-PS: Moods and Feelings Questionnaire–Short Parent version
PANAS-C: Positive and Negative Affect Schedule for Children
PHQ-8: Patient Health Questionnaire-8
SF-20: 20-item Short Form Survey
VR: virtual reality

## Data Availability

For data dictionary and deidentified individual participant data, requests can be sent to JL. Researchers whose proposed use of the data has been approved will be granted access with a signed data access agreement. Data may be used by approved researchers to better understand the analysis approach and results and to conduct additional analyses as specified.
