# Supplementary material for: Self-Guided Digital Intervention for Depression in Adolescents: Feasibility and Preliminary Efficacy Study
Source: JMIR Form Res. 2023 Nov 22;7:e43260. doi: 10.2196/43260 (PMC10701656; doi:10.2196/43260)
Supplement: Multimedia Appendix 1 [file formative_v7i1e43260_app1.doc]

## Appendix 1 - Spark Program Description

The Spark program centered around identifying values and scheduling activities, or behavioral activations (BAs), based on participants’ self-identified values[[31]](https://paperpile.com/c/p5hiXb/lZDU). These BAs were scheduled within the app and carried out in the real world. That is, participants created tickets similar to calendar events indicating an activity that they planned to do (e.g. meet up with friends). Once the participant had completed the activity, they revisited the app and indicated whether or not they had completed it. The program provided psychoeducation on the relationship between mood and behavior, guided participants in mood and activity tracking, taught mindfulness as a way to pay attention to the positive effects of behavioral activations on mood, introduced problem solving as a way to overcome obstacles in completing behavioral activations, and taught participants ways to avoid relapse and maintain gains after program completion. The mobile app was broken down into five modules (see Table 1). Module 1 contained program onboarding (e.g. introduction to the program), psychoeducation and an introduction to BA. In Module 2, after completing in-app educational activities around mood tracking and the relationships between mood and behavior, participants were recommended to complete four mood logs per day for three days for a total of 12 mood logs. In Modules 3 and 4, participants are introduced to mindfulness and problem solving, respectively. In each of these modules, participants were recommended to schedule and complete three BAs per level for a total of six recommended BAs. Only one BA was required to be completed before moving on to the next level. Beyond the one required BA, participants were allowed to schedule and complete as many BAs as they wanted. The program suggested BAs based on participants’ self-identified values that they had chosen during the program’s onboarding, or participants could schedule custom BAs. Participants were given the option in Module 5 to complete bonus BAs after learning about relapse prevention. Participants were not required to complete all the recommended mood logs or BAs to advance to the next module and could complete more than the recommended number if desired. An illustrated character within the app served as the program’s guide to encourage the user. The character, which interacted with participants via text on the screen, would suggest activities for participants to do based on values they had selected, or provide examples of how to apply skills like mindfulness breathing and mood logging.

VR experiences provided engaging psychoeducation or reinforced concepts through interactive activities. Each VR experience was associated with a specific app module, and users were prompted in the app when it was time to complete the VR portion. However, the VR experiences were not gated in the headset and could therefore be accessed at any time and completed in any order. The VR experiences delivered two immersive educational videos (modules 1 and 2), one breathing exercise, and two mindfulness exercises. This content for VR was chosen based on existing literature that suggests immersive technology may enhance learning retention and transfer of skills to the real world through psychological presence, the feeling of actually being there[*[55,56]*](https://paperpile.com/c/p5hiXb/a4qL+tlC5). Moreover teens may be more likely to engage and pay attention to this content because of the novelty of VR. Given its immersive nature, VR is a particularly viable delivery method for mindfulness and breathing exercises[[58]](https://paperpile.com/c/p5hiXb/pBki) because it blocks out a user’s surrounding environment to help them feel present in the moment and enhances the visualization of the exact exercises being taught[[57]](https://paperpile.com/c/p5hiXb/pJAi).

Participants received text messages from the research team in week 2 of the intervention period to check whether participants had any questions or technical difficulties, and in week 5 to remind participants they were in the final week of the intervention period. For data collection and integrity purposes, participants also received periodic text messages from the research team to remind them to complete their weekly PHQ-8 if they had not completed the assessment after three days. These messages were unrelated to Spark program completion (i.e. did not encourage nor reinforce progress through the program).
