# Supplementary material for: Self-Guided Digital Intervention for Depression in Adolescents: Feasibility and Preliminary Efficacy Study
Source: JMIR Form Res. 2023 Nov 22;7:e43260. doi: 10.2196/43260 (PMC10701656; doi:10.2196/43260)
Supplement: Multimedia Appendix 2 [file formative_v7i1e43260_app2.doc]

## Appendix 2. Secondary Outcomes

|  | Baseline Mean (SD) | Post  Mean (SD) | Mean Difference  Post-Pre [95%CI] | Follow-Up  Mean (SD) | Mean Difference  Follow-up - Pre  [95%CI] |
| --- | --- | --- | --- | --- | --- |
|  |  |  |  |  |  |
| CES-D | 37.89  (5.96)  n=29 | 26.81  (9.79)  n=26 | -10.85  [-15.01; -6.68]  n=26 | 25.26  (12.06)  n=27 | -13.04  [-7.58; -18.50]  n=26 |
| GAD-7 | 13.86  (4.32)  n=29 | 10.43  (4.48)  n=28 | -3.22  [-5.12; -1.32]  n=27 | 9.81  (5.05)  n=27 | -3.76  [-5.96; -1.56]  n=26 |
| PANAS-C Positive | 33.59  (7.55)  n=29 | 38.04  (10.46)  n=27 | 4.12  [0.35; 7.88]  n=26 | 37.43  (12.52)  n=28 | 3 .44  [-0.50; 7.39]  n=27 |
| PANAS-C Negative | 48.34  (10.64)  n=29 | 39.72  (10.30)  n=29 | -9.04  [-13.23; -4.84]  n=28 | 39.00  (11.81)  n=28 | -9.78  [-15.43; -4.12]  n=27 |
| BADS-SF | 16.4  (5.49)  n=30 | 22.31  (7.92)  n=29 | 5.90  [2.81; 8.99]  n=29 | 24.39  (8.27)  n=28 | 7.82  [4.30; 11.34]  n=28 |
| SF-20  Physical Functioning | 84.17  (24.79)  n=30 | 87.64  (19.12)  n=29 | 4.02  [3.44; 11.49]  n=29 | 84.82  (17.43)  n=28 | 1.79  [-4.93; 8.50]  n=28 |
| SF-20  Role Functioning | 75.83 (31.13)  n=30 | 80.17  (29.41)  n=29 | 5.17  [-3.40; 13.75]  n=29 | 79.46  (29.70)  n=28 | 5.36  [-2.27; 12.98]  n=28 |
| SF-20  Social Functioning | 62.67 (30.05)  n=30 | 70.34  (30.06)  n=29 | 8.97  [-3.66; 21.60]  n=29 | 69.29  (30.05)  n=28 | 6.43  [-7.74; 20.60]  n=28 |
| SF-20  Pain | 68.00  (22.03)  n=30 | 71.03  (21.77)  n=29 | 2.07  [-5.36; 9.50]  n=29 | 72.14  (20.61)  n=28 | 2.86  [-4.96; 10.67]  n=28 |
| SF-20  Mental Health | 35.73  (16.37)  n=30 | 47.45  (16.03)  n=29 | 11.45  [4.09; 18.81]  n=29 | 51.71  (20.77)  n=28 | 16.14  [6.42; 25.87]  n=28 |
| SF-20  Health Perception | 44.63 (20.12)  n=30 | 51.21  (24.49)  n=29 | 7.17  [0.13; 14.20]  n=29 | 54.18  (23.48)  n=28 | 11.31  [3.10; 19.52]  n=28 |
| MFQ-Parent | 12.47  (4.68)  n=17 | 6.13  (3.86)  n=16 | -5.94  [-3.44; -8.43]  n=16 | - | - |
